# Post-Transcriptional Control of LINE-1 Retrotransposition by Cellular Host Factors in Somatic Cells

**DOI:** 10.3389/fcell.2016.00014

**Published:** 2016-03-07

**Authors:** Javier G. Pizarro, Gaël Cristofari

**Affiliations:** Institute for Research on Cancer and Aging of Nice (IRCAN), Faculty of Medicine, CNRS UMR7284, INSERM U1081, University of Nice Sophia AntipolisNice, France

**Keywords:** LINE-1, retrotransposon, genome evolution, repeated sequences, retrotransposition, structural variation (SV)

## Abstract

Long INterspersed Element-1 (LINE-1 or L1) retrotransposons form the only autonomously active family of transposable elements in humans. They are expressed and mobile in the germline, in embryonic stem cells and in the early embryo, but are silenced in most somatic tissues. Consistently, they play an important role in individual genome variations through insertional mutagenesis and sequence transduction, which occasionally lead to novel genetic diseases. In addition, they are reactivated in nearly half of the human epithelial cancers, contributing to tumor genome dynamics. The L1 element codes for two proteins, ORF1p and ORF2p, which are essential for its mobility. ORF1p is an RNA-binding protein with nucleic acid chaperone activity and ORF2p possesses endonuclease and reverse transcriptase activities. These proteins and the L1 RNA assemble into a ribonucleoprotein particle (L1 RNP), considered as the core of the retrotransposition machinery. The L1 RNP mediates the synthesis of new L1 copies upon cleavage of the target DNA and reverse transcription of the L1 RNA at the target site. The L1 element takes benefit of cellular host factors to complete its life cycle, however several cellular pathways also limit the cellular accumulation of L1 RNPs and their deleterious activities. Here, we review the known cellular host factors and pathways that regulate positively or negatively L1 retrotransposition at post-transcriptional level, in particular by interacting with the L1 machinery or L1 replication intermediates; and how they contribute to control L1 activity in somatic cells.

## L1 elements contribute to the dynamics of somatic and germline human genomes

The Long INterspersed Element-1 (LINE-1 or L1) retrotransposon forms 17% of our genome (Lander et al., 2001). Most L1 copies present in the reference human genome are defective but ~100 copies could be retrotransposition-competent (Brouha et al., 2003). In addition, many polymorphic L1 elements, not included in the reference genome, also have the potential to mobilize (Beck et al., 2010; Ewing, 2015; Mir et al., 2015).

L1 elements can retrotranspose in the germline, in embryonic stem cells and in the early embryo (Kazazian et al., 1988; Garcia-Perez et al., 2007; van den Hurk et al., 2007). However, L1 retrotransposons are repressed in most tested normal somatic cells except in the brain (Coufal et al., 2009; Baillie et al., 2011; Evrony et al., 2012; Richardson et al., 2014b; Upton et al., 2015). L1 mobilization impacts human genome evolution through insertional mutagenesis and sequence transduction, which occasionally results in inherited genetic diseases (Hancks and Kazazian, 2012). Somatic retrotransposition in the brain could also contribute to the etiology of some mental disorders or disabilities, such as Rett Syndrome or Ataxia Telangiectasia, characterized by increased levels of L1 mobilization (Muotri et al., 2010; Coufal et al., 2011). Moreover, somatic L1 mobilization participates to the dynamics of many tumor genomes and can lead to driver mutations (Miki et al., 1992; Iskow et al., 2010; Lee et al., 2012; Solyom et al., 2012; Shukla et al., 2013; Helman et al., 2014; Tubio et al., 2014; Doucet-O'Hare et al., 2015; Ewing et al., 2015; Rodić et al., 2015). Besides its impact as an insertional mutagen, L1 also triggers other forms of genomic alterations such as DNA double-strand breaks or chromosomal translocations, and these activities could participate to normal aging or tumorigenesis (Wallace et al., 2008; Lin et al., 2009; Belancio et al., 2010). Finally, the L1 machinery also drives the retrotransposition of Short INterspersed Elements (SINEs) and the formation of processed pseudogenes (Esnault et al., 2000; Dewannieux et al., 2003).

L1 elements and their host have co-evolved: L1s use the cellular machinery for their own replication, while the host cell has evolved multiple defense mechanisms limiting L1 deleterious effects. Silencing L1 expression, through CpG DNA methylation and histone modifications is a major repressive mechanism, which prevents the accumulation of mutagenic events (Bourc'His and Bestor, 2004; Castro-Diaz et al., 2014; Jacobs et al., 2014). Here we review post-transcriptional cellular pathways, which regulate positively or negatively L1 retrotransposition in somatic cells, in particular by interacting with the L1 machinery or L1 replicative intermediates.

## L1 replication is mediated by a ribonucleoprotein particle (RNP) and target-primed reverse transcription (TPRT)

**Figure 1:**
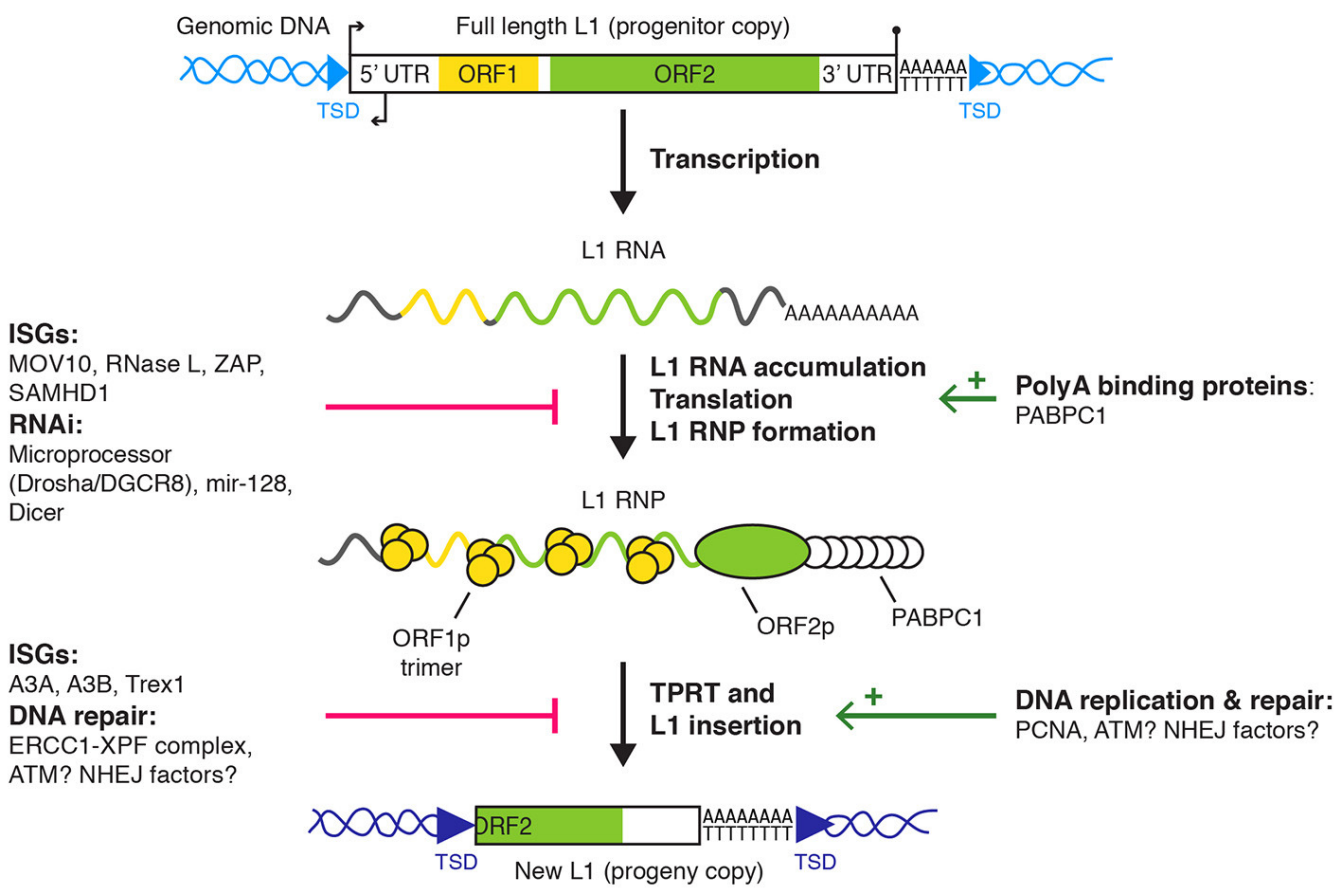
**L1 retrotransposition and cellular regulators**. L1 replication starts with L1 transcription into a full length bicistronic L1 mRNA, its translation into ORF1p and ORF2p, and the assembly of an L1 RNP. For the sake of simplicity, the recently described antisense ORF0 in the 5′ UTR is not depicted (Denli et al., 2015). The L1 RNP accumulates in stress granules and at least a fraction of it is imported to the nucleus (not shown) where target-primed reverse transcription (TPRT) occurs at the target DNA site. Finally, L1 insertion is resolved by an unknown mechanism (see main text for details). Only L1 regulators with a defined target/mechanism are depicted. Small broken arrows, L1 sense and antisense promoter activities; lollipop, L1 polyadenylation signal; light and dark blue arrowheads, target sites of L1 progenitor and progeny copies, respectively; red bars, negative regulation; green arrows, positive regulation. TSD, target site duplication; UTR, untranslated region; RNP, ribonucleoprotein particle; ISG, interferon-responsive genes; RNAi, RNA interference.

An active L1 retrotransposon comprises a 5′ untranslated region (UTR), two open reading frames (ORF1 and ORF2) separated by a short inter-ORF spacer and a 3′ UTR (Figure 1). An antisense ORF0 of unknown function has also been recently described in the 5′ UTR (Denli et al., 2015). As a consequence of the reverse transcription and integration mechanism, L1 sequence ends with a poly(dA) stretch and is flanked by target site duplications (TSD) of variable size. The 5′ UTR contains RNA polymerase II sense and antisense promoters (Swergold, 1990; Speek, 2001; Nigumann et al., 2002). The translation of the bicistronic L1 mRNA by an unconventional mechanism produces two proteins, named ORF1p and ORF2p (Alisch et al., 2006; Dmitriev et al., 2007). ORF1p is a 40 kDa RNA-binding protein, forming trimers and with nucleic acid chaperone activity (Martin, 1991; Holmes et al., 1992; Martin and Bushman, 2001; Martin et al., 2003; Khazina et al., 2011). ORF2p is a ~150 kDa protein with endonuclease (EN) and reverse transcriptase (RT) activities, which are critical for L1 retrotransposition (Mathias et al., 1991; Feng et al., 1996; Moran et al., 1996). ORF2p also contains a C-terminal cysteine-rich region, potentially contributing to its RNA binding capability (Piskareva et al., 2013). ORF1p and ORF2p bind the L1 mRNA to form a ribonucleoprotein particle (RNP), considered as the core of the L1 replicative complex (Hohjoh and Singer, 1996; Kolosha and Martin, 1997; Kulpa and Moran, 2005, 2006; Doucet et al., 2010; Goodier et al., 2010). This assembly occurs preferentially *in cis* (Esnault et al., 2000; Wei et al., 2001; Kulpa and Moran, 2006), through binding of ORF2p to the L1 RNA poly(A) sequence (Doucet et al., 2015). L1 RNPs accumulate in cytoplasmic foci, which colocalize with stress granules (Goodier et al., 2007, 2010; Doucet et al., 2010). The functional importance of these cytoplasmic complexes remains to be elucidated. Although cell division seems to promote retrotransposition, it is not absolutely required (Kubo et al., 2006; Shi et al., 2007; Xie et al., 2013). Thus, access of L1 RNPs to chromatin can occur independently of mitotic nuclear envelope breakdown through an unknown nuclear import mechanism.

New L1 copies are directly synthesized and inserted in the genome by a process called TPRT (Luan et al., 1993; Feng et al., 1996; Cost et al., 2002; Christensen et al., 2006). During TPRT, ORF2p binds and nicks a consensus sequence of the form 5′-TTTT/A-3′ in the genomic DNA (Feng et al., 1996). This cleavage, potentially followed by additional processing steps, exposes a single-stranded T-rich DNA stretch able to partially or completely anneal to the L1 RNA poly(A) tail and to prime ORF2p-mediated reverse transcription (Kulpa and Moran, 2006; Monot et al., 2013; Viollet et al., 2014). A possible second nick, generally few nucleotides downstream of the first one, allows priming and synthesis of the second DNA strand. Finally, the L1 DNA ends are filled in and sealed, creating TSD (Luan et al., 1993; Feng et al., 1996; Cost et al., 2002). The molecular actors involved in these late stages are unknown. This process is frequently abortive, resulting in 5′ truncated L1 copies.

## L1 retrotransposition is regulated by cellular factors at multiple levels

L1 activity is regulated at multiple stages of the L1 retrotransposition cycle (Figure 1). We focus here on post-transcriptional mechanisms and their molecular effectors acting in human or mammalian somatic cells and interacting with components of the L1 RNP or with L1 replication intermediates. L1 regulation in the germline, notably by Piwi-interacting RNA (piRNA), has been reviewed elsewhere (Zamudio and Bourc'his, 2010; Crichton et al., 2014) and is not detailed in the present article.

### Proteomic studies have revealed cellular partners of L1 RNPs and potential novel regulators of L1 retrotransposition

#### Overview

**Figure 2:**
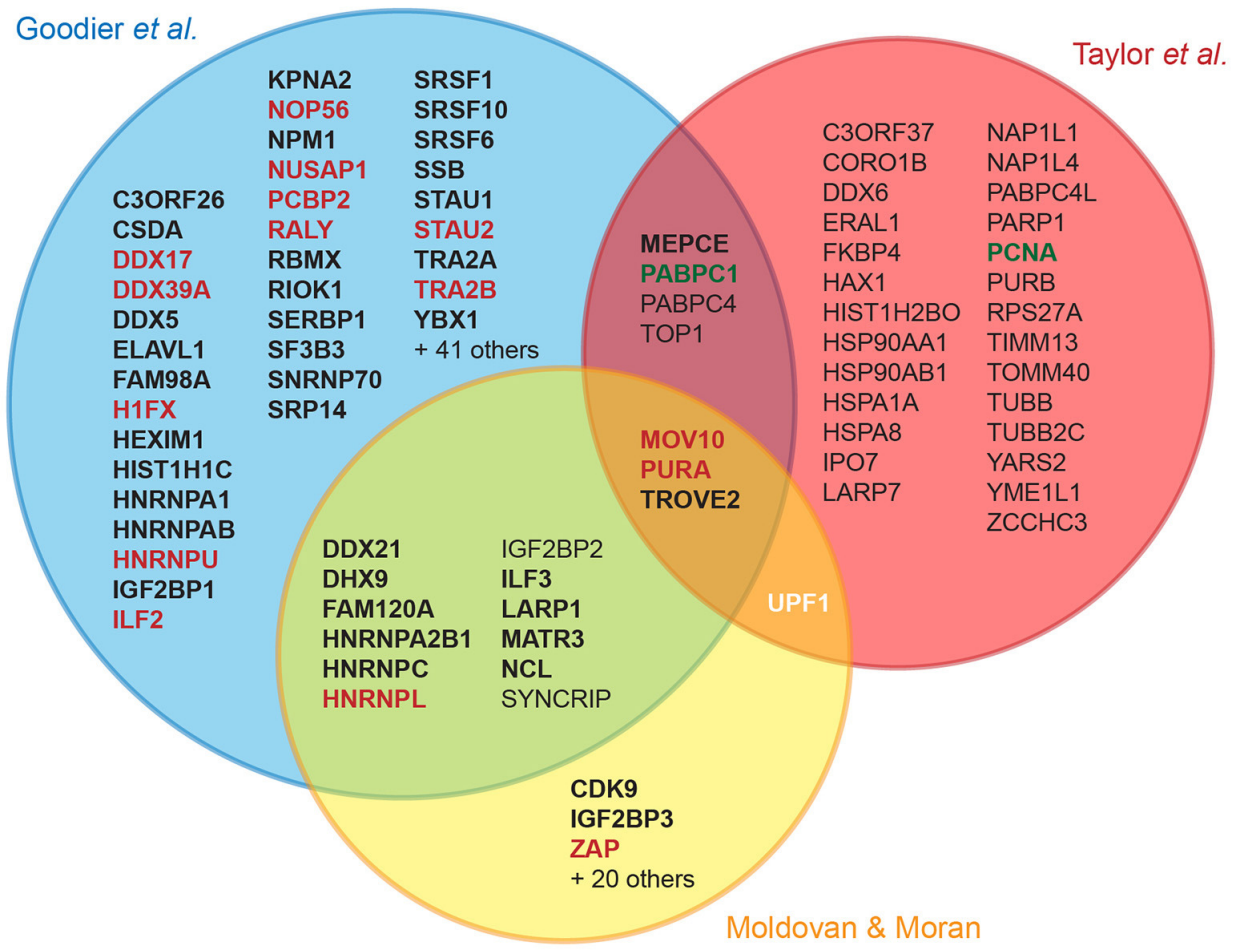
**Cellular L1 interactors discovered in recent proteomic studies**. The Venn diagram displays the overlap between three major proteomic studies designed to identify L1 cellular partners (Goodier et al., 2013; Taylor et al., 2013; Moldovan and Moran, 2015). For the sake of simplicity, the overlap with a more limited fourth study is not shown, but includes NCL and HNRNPL (Peddigari et al., 2013). For hits found in a single study, only those confirmed by coimmunoprecipitation (coIP) or by quantitative mass-spectrometry are depicted. Bold, confirmed by coIP; red and green, L1 negative and positive regulators, respectively; white, potential dual role: UPF1 knockdown decreases overall L1 retrotransposition but increases L1 RNA levels, suggesting that it could act at several stages with opposing effects (Taylor et al., 2013). Upf1 overexpression is not impacting retrotransposition (Moldovan and Moran, 2015).

Several recent studies have identified cellular partners of L1 RNPs through tagging of ORF1p, ORF2p or L1 RNA, followed by affinity chromatography and mass-spectrometry (Goodier et al., 2013; Peddigari et al., 2013; Taylor et al., 2013; Moldovan and Moran, 2015). These experimental efforts differ by the cell line, the L1 clone, the tagged component in the complex and the chromatography method used, but eventually lead to a number of common host factors (Figure 2). It should be underlined that only a fraction of the hits has been validated by co-immunoprecipitation, and only a single study used quantitative mass-spectrometry to measure the specific enrichment of the detected proteins upon elution (Taylor et al., 2013). A first step toward functional characterization generally involves retrotransposition assays in cultured cells upon depletion or overexpression of the tested factor. The outcome of these genetic assays allows a first classification into positive or negative regulators. However, many binding partners only modestly impact the levels of L1 retrotransposition in these assays, or have pleiotropic effects preventing unambiguous interpretation. With few exceptions, the majority of the tested factors are RNA-binding proteins, which copurify with ORF1p through an indirect RNA bridge, colocalize with L1 RNPs in stress granules, and inhibit L1 retrotransposition.

#### Limitations

Due to the scarcity of L1 endogenous complexes in cells, all proteomic studies rely on the overexpression of engineered L1 constructs. It is conceivable that: (i) some of the discovered partners become associated with L1 components as a result of L1 overexpression beyond physiological levels. (ii) L1 RNP stoichiometry is altered; (iii) the retrotransposition reporter cassette, which contains an intron, modifies L1 RNA cellular processing, and thus its binding partners.

### Positive regulators of L1 retrotransposition

#### Poly(A) binding proteins act in L1 RNP assembly or trafficking

Poly(A) binding proteins (PABPs) bind mRNA poly(A) tails and are involved in mRNA stability and translation initiation (Goss and Kleiman, 2013). Short hairpin RNA (shRNA)-mediated knockdown of PABPC1, reduces L1 retrotransposition with minimal effects on L1 RNA and proteins accumulation, or poly(A) tail length (Dai et al., 2012). This effect is associated with reduced L1 RNP levels and reduced nuclear accumulation of this complex, suggesting a possible—direct or indirect—role of PABPC1 in the assembly or the subcellular trafficking of the L1 RNP. Consistently, PABPC1 associates with the L1 RNP in an RNA-dependent manner, they colocalize in stress granules (Goodier et al., 2013; Taylor et al., 2013), and moderate PABPC1 overexpression stimulates retrotransposition (Dai et al., 2012). Other PABPs have been found to associate with the L1 RNA (PABPN1, PABPC4) but addressing their specific role in L1 retrotransposition has been hampered by pleiotropic effects, or not yet tested (Dai et al., 2012; Goodier et al., 2013; Taylor et al., 2013).

#### PCNA is a cofactor of TPRT

PCNA is a DNA sliding clamp acting as a processivity factor for many DNA polymerases during DNA replication or DNA damage repair (Moldovan et al., 2007). ORF2p binds PCNA through a PCNA-interacting protein (PIP) box, located between the EN and RT domains of ORF2p (Taylor et al., 2013). Mutations in ORF2p PIP box disrupt PCNA-ORF2p interaction and inhibit L1 retrotransposition. Interestingly, ORF2p mutations abrogating its EN or RT activity also disrupt PCNA-ORF2p interaction, suggesting that PCNA binding to ORF2p occurs downstream or concomitantly with TPRT.

#### Proline-directed protein kinase(s) regulate(s) ORF1p function

ORF1p contains several (S/T)-P putative phosphorylation sites for proline-directed protein kinases (PDPKs), such as mitogen-activated protein kinases and cyclin-dependent kinases. Mutations of S18, S27, T203, and T213, which are potential PDPK targets, decrease L1 retrotransposition; and these residues were found phosphorylated by mass-spectrometry in human cells (Cook et al., 2015). Interestingly, several protein kinases associate with the L1 RNP (Goodier et al., 2013; Taylor et al., 2013; Moldovan and Moran, 2015), however it remains to be demonstrated if one or several of them might directly target ORF1p. Interestingly, S18/S27 sites in ORF1p are required for binding by Pin1 prolyl isomerase (Cook et al., 2015), suggesting a scenario in which binding of Pin1 promotes ORF1p conformational change, which could affect its stability, activity or localization, or its subsequent ability to be dephosphorylated (Yeh et al., 2004; Liou et al., 2011).

### Cellular pathways inhibiting L1 retrotransposition at post-transcriptional level

#### RNA interference pathways prevent the accumulation of L1 RNA

L1 RNA serves both as an mRNA to produce the L1 machinery and as a template for reverse transcription. Multiple RNA interference (RNAi) pathways act in somatic or embryonic cells to prevent the accumulation of L1 RNA, and eventually retrotransposition.

First, the Microprocessor complex (Drosha/DGCR8), a major nuclear complex implicated in microRNA (miRNA) biosynthesis through pri-miRNA processing, is also able to bind L1 RNA *in vivo*, to reduce its abundance and to limit L1 retrotransposition. In addition, it can cleave various L1 RNA fragments derived from the L1 5′ UTR region *in vitro*, indicating that L1 RNA can be a direct Microprocessor substrate (Heras et al., 2013, 2014). Moreover, the miRNA pathway could also act downstream of Microprocessor to inhibit retrotransposition. Indeed, miR-128 in complex with the Argonaute (Ago) protein binds the L1 RNA in the ORF2 region leading to L1 transcript degradation (Hamdorf et al., 2015).

Second, the combined expression of sense and antisense L1 transcripts driven by L1 5′ UTR promoters reduces L1 RNA stability and L1 retrotransposition (Yang and Kazazian, 2006). This process is associated with the synthesis of rasiRNA (repeat-associated small interfering RNA) consistent with a possible processing of L1 RNA duplexes, and is modestly inhibited by Dicer knockdown, suggesting an additional layer of L1 repression mediated by siRNA mechanisms. In agreement with a role of RNAi pathways in somatic L1 regulation, L1 RNPs tend to accumulate in stress granules where they colocalize with several RNAi factors and often interact with them (Goodier et al., 2007, 2013).

#### Innate immunity and interferon response pathways

The cellular innate immune response is one of the first lines of defense against a broad range of viral infections. It involves cellular factors with antiviral activities, among which the interferon (IFN) response pathway plays a central role (MacMicking, 2012; Ivashkiv and Donlin, 2014). This pathway leads to the activation of IFN-stimulated genes (ISG) acting as effectors and reinforcing IFN-signaling itself. A significant proportion of ISG are viral restriction factors (MacMicking, 2012), which also appear to counteract L1 retrotransposition (Goodier et al., 2015), and are described below.

Upon overexpression, several members of the APOBEC3 (A3) cytidine deaminase family inhibit L1 retrotransposition (A3A, A3B, A3C and A3F) (Bogerd et al., 2006; Chen et al., 2006; Muckenfuss et al., 2006; Stenglein and Harris, 2006; Kinomoto et al., 2007; Niewiadomska et al., 2007). A3A is a nuclear protein predominantly expressed in peripheral blood mononuclear cells (PBMCs) and is induced by IFN-β (Chen et al., 2006; Muckenfuss et al., 2006; Stenglein et al., 2010). A3A-mediated L1 inhibition depends on A3A deaminase activity and on the subsequent processing of the deaminated DNA by uracil DNA glycosylase (UNG) and apurinic/apyrimidinic endonuclease (APE) (Richardson et al., 2014a). A3B is also a nuclear protein. It is endogenously expressed in embryonic stem cells, in induced-pluripotent stem cells and in a number of cancer cell lines. Its depletion stimulates L1 retrotransposition (Wissing et al., 2011; Marchetto et al., 2013); however, catalytically dead A3B mutants still inhibit L1 retrotransposition (Wissing et al., 2011). Thus, the mechanism by which A3B represses L1 mobilization remains unknown. Similarly, reducing the expression of A3C moderately increases retrotransposition in cancer cell lines that express detectable levels of endogenous A3C (Muckenfuss et al., 2006). As for A3B, A3C- and A3F-mediated L1 repression is deaminase-independent (Muckenfuss et al., 2006; Stenglein and Harris, 2006; Kinomoto et al., 2007; Horn et al., 2014). A3C might interfere with L1 reverse transcription or the activity of ORF2p in the L1 RNP (Horn et al., 2014).

Several other ISG products, such as MOV10, ZAP or RNase L, limit L1 replication by limiting L1 RNA accumulation. The RNA helicase MOV10 robustly copurifies with the L1 complex, colocalizes with L1 RNPs in stress granules, reduces L1 RNA half-life, and ultimately strongly inhibits retrotransposition (Arjan-Odedra et al., 2012; Goodier et al., 2012, 2013; Li et al., 2013; Taylor et al., 2013; Moldovan and Moran, 2015). Similarly, the zinc-finger antiviral protein (ZAP) associates with the L1 RNP and accumulates with it in stress granules (Goodier et al., 2015; Moldovan and Moran, 2015). Its overexpression reduces full-length L1 RNA levels, and L1 retrotransposition levels. ZAP zinc finger domain is necessary and sufficient for its anti-L1 activity. Inversely, knocking down endogenous ZAP increases L1 retrotransposition. The ribonuclease L (RNase L) degrades L1 RNA and inhibits retrotransposition although no association or colocalization was detected with the L1 RNP (Zhang et al., 2014). Other ISGs with known viral restriction activities (e.g., *BST2, ISG20, MAVS*, and *MX2*) are also strong inhibitors of L1 retrotransposition (Goodier et al., 2015), but their mechanism of action has not yet been explored.

Finally, *SAMHD1* and *TREX1* are ISGs involved in a negative feedback loop, acting as repressors of the interferon response itself. Loss-of-function mutations in these genes lead to the Aicardi-Goutières syndrome, an autoimmune disease. Both factors inhibit L1 retrotransposition (Stetson et al., 2008; Zhao et al., 2013). Trex1 (Three-prime-repair exonuclease 1) is an abundant 3′-5′ DNA exonuclease and its overexpression inhibits engineered L1 retrotransposition in cultured cells (Stetson et al., 2008). Trex1-deficient cells accumulate ssDNA fragments derived from various retroelements including L1, suggesting that Trex1 can metabolize reverse transcribed L1 cDNA (Stetson et al., 2008). SAMHD1 (SAM Domain And HD Domain 1) impairs lentivirus replication in non-dividing cells by depleting the intracellular pool of dNTPs and thereby inhibiting reverse transcription (Lahouassa et al., 2012). In contrast, SAMHD1 inhibits L1 retrotransposition in dividing cells, through a dNTPase-independent mechanism, which might directly affect ORF2p levels, and thus inhibit L1 reverse transcription (Zhao et al., 2013).

#### DNA repair pathways

EN-mediated cleavage of the target DNA or other TPRT intermediates could lead to DNA double-strand break (DSB) or DNA lesion signaling, and activation of subsequent DNA repair pathways. Conversely, these cellular processes could also participate in the resolution of L1 integration, through L1 second strand DNA synthesis or DNA ligation.

The role of DSB signaling and non-homologous end-joining (NHEJ) pathways remains controversial. Ataxia-telangiectasia mutated (ATM) protein, a kinase activated upon DSB, was initially proposed to be required for L1 retrotransposition and L1-induced DSBs (Gasior et al., 2006; Wallace et al., 2013). However, independent studies using ATM-deficient mice or human cell models rather suggest that ATM is a repressor of retrotransposition (Coufal et al., 2011). Similarly, knocking out NHEJ genes (e.g., Ku70/80, DNA Ligase IV or Artemis) decreases L1 retrotransposition in chicken cells (Suzuki et al., 2009). However, loss-of-function of DNA-PKcs or DNA Ligase IV in mammalian cells does not impair L1 retrotransposition (Coufal et al., 2011), indicating that NHEJ is not absolutely required for L1 retrotransposition. An interesting possibility could be that DSB signaling and repair pathways compete with the L1 machinery or other cellular factors for the resolution of L1 insertion during—or after—cDNA synthesis, leading to 5′ truncated insertions (Zingler et al., 2005; Suzuki et al., 2009; Coufal et al., 2011).

Other DNA repair pathways can also antagonize L1 replication. The ERCC1-XPF complex, which plays a role in nucleotide excision, base excision and interstrand crosslink repair pathways is a potent inhibitor of L1 retrotransposition (Gasior et al., 2008). ERCC1-XPF is an endonuclease able to specifically cleave DNA at junctions between single-stranded and double-stranded regions, a predicted structure produced by the TPRT process. Thus, it has been hypothesized that ERCC1-XPF might cut off L1 cDNA at the target site during reverse transcription.

## Open questions for the future

- How is unspliced L1 RNA exported to the cytosol and the L1 RNP imported back to the nucleus?
- How many distinct L1 RNP forms exist in the cell and throughout the L1 replication cycle?
- Do L1 components have a life outside of the L1 RNP and retrotransposition?
- How is L1 RNP assembly regulated?
- Does L1 component accumulation in stress granules reflect a host defense mechanism or an intermediate step during retrotransposition?
- Which restriction factors are the dominant ones and do they cooperate?

## Author contributions

JGP drafted the manuscript. GC revised the manuscript.

## Funding

GC is funded by the Fondation ARC pour la recherche sur le cancer, the European Research Council (ERC-2010-StG 243312, RETROGENOMICS), the French Government (National Research Agency, ANR) through the “Investments for the Future” (LABEX SIGNALIFE, ANR-11-LABX-0028-01), and the Fondation pour la Recherche Médicale (FRM DEP20131128533).

### Conflict of interest statement

The authors declare that the research was conducted in the absence of any commercial or financial relationships that could be construed as a potential conflict of interest.
